# Clinical Significance of Non-Coding RNA Regulation of Programmed Cell Death in Hepatocellular Carcinoma

**DOI:** 10.3390/cancers15164187

**Published:** 2023-08-21

**Authors:** Wuyu Chen, Minghao Ruan, Minghao Zou, Fuchen Liu, Hui Liu

**Affiliations:** The Third Department of Hepatic Surgery, Eastern Hepatobiliary Surgery Hospital, Naval Medical University, Shanghai 200438, China; chenwuyu1999@163.com (W.C.); mingm0706@163.com (M.R.);

**Keywords:** non-coding RNA, hepatocellular carcinoma, programmed cell death, prognosis, drug resistance

## Abstract

**Simple Summary:**

Hepatocellular carcinoma is a prevalent and aggressive liver tumor. Non-coding RNAs that do not participate in protein synthesis but regulate gene expression have been found to influence programmed cell death. Understanding the clinical significance of non-coding RNA regulation of programmed cell death in hepatocellular carcinoma, such as patient diagnosis and prognosis, drug resistance, and drug side effects, can provide valuable insights into the diagnosis and treatment of hepatocellular carcinoma.

**Abstract:**

Hepatocellular carcinoma (HCC) is a widely prevalent and malignantly progressive tumor. Most patients are typically diagnosed with HCC at an advanced stage, posing significant challenges in the execution of curative surgical interventions. Non-coding RNAs (ncRNAs) represent a distinct category of RNA molecules not directly involved in protein synthesis. However, they possess the remarkable ability to regulate gene expression, thereby exerting significant regulatory control over cellular processes. Notably, ncRNAs have been implicated in the modulation of programmed cell death (PCD), a crucial mechanism that various therapeutic agents target in the fight against HCC. This review summarizes the clinical significance of ncRNA regulation of PCD in HCC, including patient diagnosis, prognosis, drug resistance, and side effects. The aim of this study is to provide new insights and directions for the diagnosis and drug treatment strategies of HCC.

## 1. Introduction

**Figure 1:**
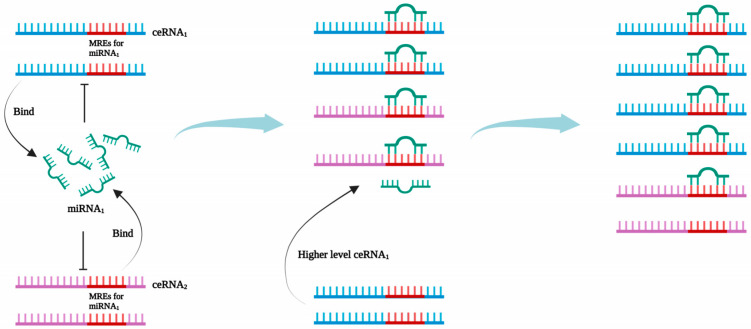
Competing endogenous (ceRNA) network mechanism. ceRNA_1_ and ceRNA_2_ possess the same miRNA-responsive elements (MREs), thus allowing them to bind miRNA_1_ competitively. The increased expression of ceRNA_1_ levels allows for a competitive binding with a high number of miRNA_1_ molecules. This leads to a reduction in the ceRNA_2_ that is inhibited by miRNA_1_, resulting in increased expression of ceRNA_2_.

Hepatocellular carcinoma (HCC) is a prevalent malignancy with a high global incidence rate [1]. It is the third most prevalent cause of mortality globally, accounting for a substantial proportion of over 8% of all cancer-related fatalities [2]. In current clinical settings, the predominant approach for managing patients with HCC is surgical interventions, particularly radical hepatectomy. This approach continues to be favored despite notable advancements in the diagnosis and treatment of HCC [3]. The insidious onset of HCC often leads to a delayed diagnosis, with a majority of patients being identified during the middle and late stages of the disease. This unfortunate circumstance contributes to the missed opportunity for timely surgical interventions. Therefore, it is imperative to devise novel strategies for the prevention and pharmacological management of HCC. Non-coding RNAs (ncRNAs) are a class of RNA molecules that do not possess the ability to encode proteins. However, they play a crucial role in the regulation of various cellular processes, thereby contributing to the maintenance of cellular homeostasis [4]. In the category of ncRNAs, three distinct types have emerged as particularly influential: microRNA (miRNA), long non-coding RNA (lncRNA), and circular RNA (circRNA). miRNAs are a class of endogenous RNA molecules, approximately 22 nucleotides in length, that have been found to play crucial regulatory roles in animals and plants. These molecules can target specific messenger RNAs (mRNAs) for either cleavage or translational repression, thereby exerting control over gene expression [5]. lncRNAs represent a distinct category of RNA molecules characterized by their extended nucleotide sequence exceeding 200 units. These molecules exert regulatory control over gene expression through diverse mechanisms, such as direct interactions with DNA, RNA, or proteins. Additionally, lncRNAs influence various cellular processes, including chromatin modification, transcriptional regulation, RNA splicing, transport, and degradation. circRNAs exhibit a distinctive circular conformation, established via covalent bonding, and are devoid of the conventional 5’-terminal cap and 3’-terminal poly(A) tail [6]. They play a pivotal role in shaping gene expression by modulating miRNA activity, regulating transcription, and interfering with splicing processes [7]. Moreover, the hypothesis of competing endogenous RNA (ceRNA) presents a significant post-transcriptional regulatory network, which encompasses an intricate interaction among circRNAs, lncRNAs, and various other RNAs that possess miRNA binding sites. The regulatory role of these ncRNAs and protein-coding mRNAs in gene expression has been elucidated through their ability to function as natural miRNA sponges via miRNA response elements. This phenomenon allows these ncRNAs and mRNAs to engage in competitive interactions with miRNAs, thus affecting the expression of mRNAs and the subsequent protein levels [8,9] (Figure 1).

Recent progress shows that ncRNAs play a vital role in the dysregulation, initiation, and progression of HCC. This involves many aspects of HCC, such as tumor microenvironment, epithelial–mesenchymal transition, tumor invasiveness, and tumor metabolism [10,11]. It is noteworthy to highlight that ncRNAs have been extensively associated with the intricate control of programmed cell death (PCD) in HCC. These regulatory processes encompass multiple forms of cell death, such as autophagy, ferroptosis, and other related pathways. These pathways play a crucial role in determining the ultimate cell fate. The dysregulation of PCD has been shown to have a significant impact on treatment outcomes and prognosis in patients [12,13]. Hence, it is of paramount importance to understand the clinical implications associated with the regulation of ncRNA in PCD within HCC.

This review aims to provide a relatively comprehensive analysis of the current research on the significance of ncRNA regulation of PCD in terms of diagnosis, prognosis, drug resistance, and drug side effects in HCC patients. The roles of three types of ncRNAs, namely miRNAs, lncRNAs, and circRNAs, will be the primary focus of this analysis. Our ultimate goal is to offer new insights that can facilitate the development of more effective therapeutic strategies for HCC.

## 2. Diagnosis and Survival Prognosis

Alpha-fetoprotein (AFP) serves as the conventional biomarker employed for the early identification of HCC. Recent investigations have revealed that utilizing AFP in medical settings has sparked debate due to its limited sensitivity and specificity in HCC screening. Several studies have reported the absence of elevated AFP levels in patients diagnosed with HCC. In contrast, elevated levels of AFP have been documented in individuals afflicted with illnesses such as hepatic cirrhosis, cholangiocarcinoma, or other neoplasms not associated with HCC [14]. Consequently, the guidelines established by the American Association for the Study of Liver Diseases in 2010 removed the suggestion of employing AFP as a screening tool for HCC [15]. In accordance with the 2012 guidelines established by the European Association for the Study of the Liver, it was determined that AFP lacks both sensitivity and specificity as a diagnostic tool [16]. The functional role of miRNAs in HCC diagnosis has been verified in many studies. The plasma of patients with low AFP contained elevated levels of miR-10b, miR-21, and miR-182, which could be used as early diagnostic markers for HCC [17]. In a recent study conducted by Wang et al., it was demonstrated that the levels of miR-122, miR-148a, and miR-1246 in serum exosomes exhibit a notable increase in individuals with HCC compared to both liver cirrhosis patients and individuals in the normal control group. This finding suggests that these specific miRNAs may serve as potential biomarkers for distinguishing HCC from other liver conditions [18]. In contrast to miRNAs, lncRNAs exhibit greater stability in their expression levels and are more readily detectable in various bodily fluids [19]. Thus, lncRNAs combined with other molecules, specifically HCC biomarker AFP, are more likely to be a desirable HCC diagnosis method. For instance, the combination of serum lncRNAs UCA1 and WRAP53 with AFP achieves sensitivity up to 100% in HCC diagnosis [20]. Furthermore, it has been shown that circCDYL exhibits a notable and selective expression pattern in the initial stages of HCC tissue [21]. Recent studies have shown that ncRNAs can also be detected in saliva, urine, ascites, and bile. This is expected to provide a more convenient and non-invasive diagnostic method for HCC and provide strong support for the early detection and treatment of liver cancer [22].

The adverse prognosis of HCC is still a severe problem for decades [23]. Numerous therapeutic tools have been subjected to extensive investigation in medical research, yielding promising outcomes. However, it is important to note that the efficacy of HCC treatment remains uncertain, posing a significant challenge [24]. Several studies documented evidence that ncRNAs play a crucial role in tumor proliferation, migration, and invasion [25]. Thus, an RNA-based targeted approach for diagnosing and treating HCC or predicting the outcome may be potentially valuable in the future [26,27]. So far, the methods of analyzing the therapeutic effect, especially in the long-term efficacy of HCC, are overall survival and evolution-free survival (DFS, disease-free survival; RFS, relapse-free survival; PFS, progression-free survival) [28]. The survival time estimation serves as a highly intuitive parameter that effectively reflects the treatment response in HCC patients (Table 1).

Understanding the role of specific miRNAs in HCC further elucidates their impact on survival outcomes. The role of miR-101 in cellular processes involves the inhibition of autophagy, induction of apoptosis, and suppression of tumorigenicity [29,30]. Zhang et al. noted that downregulation of miR-101 was associated with adverse OS of patients with HCC in a cohort study [31]. Another miRNA, miR-122, targets the downregulation of the anti-apoptotic gene Bcl-w, triggering apoptosis and predicting better OS and DFS in HCC patients [32,33]. In contrast, silencing of miR-221 induces apoptosis and G2/M phase arrest and inhibits cellular proliferation by upregulating p53 and downregulating Bcl-2 in HCC cells, leading to increased OS, RFS, and PFS in patients [34]. Furthermore, a study conducted by Lu et al. observed that a higher level of miR-23a-3p in HCC patients correlated with poorer overall OS and RFS. The phenomenon can be attributed to the epigenetic mechanism of downregulation, specifically targeting the process of ferroptosis through the inhibition of acyl-CoA synthetase long-chain family member 4 (ACSL4) [35].

lncRNA PVT1 plays an essential role in modulating HCC cell proliferation and apoptosis by recruiting the enhancer of zeste homolog 2 (EZH2). Patients with high levels of PVT1 generally experience lower survival rates [36,37]. In contrast, HCC patients who possess a reduced amount of lncRNA PLAC2 demonstrate notably reduced OS rates compared to individuals with elevated levels of PLAC2 expression. This is attributed to the ability of PLAC2 to upregulate p53 and induce apoptosis in HCC cells [38]. Furthermore, the downregulation of lncRNA HCG18 has been observed to have an impact on the control of glutathione peroxidase 4 (GPX4)-mediated inhibition of ferroptosis through the sequestration of miR-450b-5p. This molecular mechanism is correlated with a more favorable prognosis in HCC patients [39].

circRNAs often contribute to poorer prognosis in HCC patients and are frequently associated with a decrease in HCC cell apoptosis. The downregulation of circ_0021093 significantly inhibits HCC cell proliferation by promoting cell apoptosis and impairing cell migration and invasion capabilities. Overexpression of circ_0021093 forecasts a reduced survival rate by targeting the miR-766-3p/metastasis-associated protein 3 (MTA3) pathway in HCC [40]. circRNA circ-FOXP1, induced by gene SOX9, revealed a cell-protective role by sequestering miR-875-3p and miR-421, thus mitigating apoptosis. HCC patients with elevated circRNA circ-FOXP1 expression exhibited significantly reduced OS rates [41]. However, the knockdown of circRNA circMDK has been exhibited to enhance the apoptosis ratio of HCC cells, thereby suggesting its potential role as a tumor suppressor. In accordance with this finding, the upregulation of circMDK has been correlated with a decreased 5-year survival rate in HCC patients [27].

As for tRNAs, they are a kind of ncRNA that play a key role during protein translation [42]. An expanding body of empirical evidence has substantiated the association between tRNA modification and tumor progression. For instance, Huang et al.’s study suggested that METTL1-mediated m7G tRNA modification inhibited HCC cell apoptosis and made OS worse in HCC patients [43]. In another study, low expression of tRNA methyltransferase 5 (TRMT5), which catalyzes the m1G37 modification of mitochondrial tRNAs in HCC cells, could increase cell apoptosis and exhibit better overall survival in HCC patients [44].

**Figure 2:**
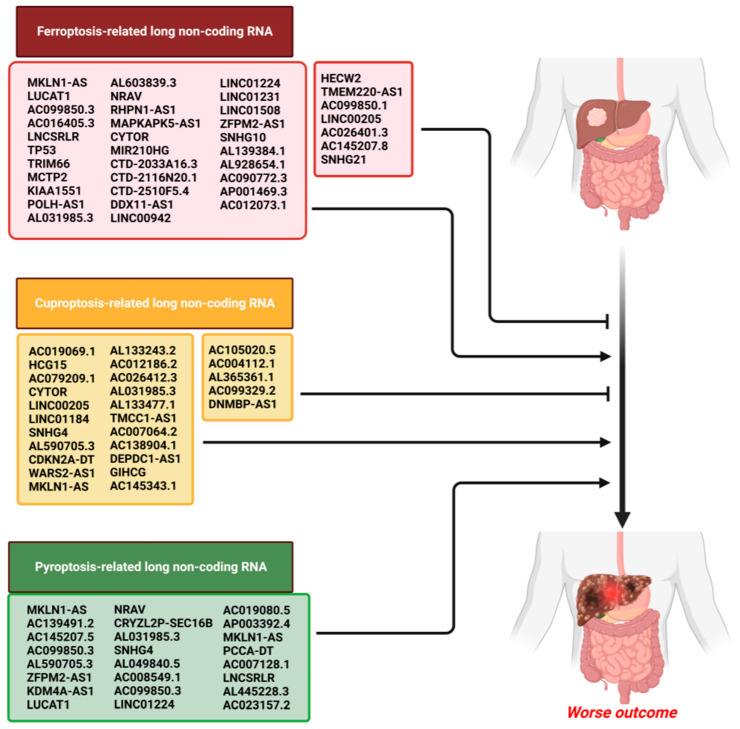
lncRNAs associated with PCD in the construction of survival prognostic models.

In light of the progress achieved with bioinformatics techniques, lncRNAs associated with PCD have become widely employed in the development of prognostic models for assessing the survival outcomes of HCC patients. Several survival prognostic models are currently constructed via PCD-related lncRNAs from online databases. These provide new insights into exploring the correlation between HCC and PCD and assessing the prognosis of HCC [45,46,47,48,49,50,51,52,53,54,55,56,57,58,59,60,61] (Figure 2).

The elucidation of ncRNAs through a comprehensive analysis of clinical samples and fundamental experiments has shed light on a potentially effective pattern for managing and predicting HCC. Also, the implementation of precision medicine, customized for individual patient’s unique conditions, has emerged as the predominant trend in the field. The investigation of the expanding collection of ncRNAs linked to HCC represents an exciting opportunity for scientific inquiry.

**Table 1:** Prognosis of ncRNAs and HCC patient.

ncRNAs	Targets	Effect	Mechanism	Refs.
miR-101	-	Pro-apoptosis Anti-autophagy Better OS	The function of miR-101 is inhibiting autophagy, inducing apoptosis, and suppressing tumorigenicity. Downregulation of miR-101 associated with poorer OS of patients.	[29,30,31]
miR-122	↓Bcl-w	Pro-apoptosis Better OS and DFS	miR-122 targets the downregulation of the anti-apoptotic gene Bcl-w, thereby triggering apoptosis and predicting better OS and DFS in HCC patients.	[32,33]
miR-221	↓P53 ↑Bcl-2	Anti-apoptosis Poorer OS, RFS, and PFS	Silencing of miR-221 induces apoptosis and G2/M phase arrest and inhibits cellular proliferation by upregulating P53 and downregulating Bcl-2 in HCC cells, leading to increased OS, RFS, and PFS in patients.	[34]
miR-23a-3p	↓ACSL4	Anti-ferroptosis Poorer OS and RFS	An elevated level of miR-23a-3p in HCC patients is associated with OS and RFS, potentially due to the epigenetic downregulation of ferroptosis by inhibiting ACSL4.	[35]
lncRNA PVT1	↑EZH2	Anti-apoptosis Poorer OS	PVT1 recruits EZH2 to modulate HCC cell proliferation and apoptosis, leading to lower survival rates in patients with high PVT1 levels.	[36,37]
lncRNA PLAC2	↑P53	Pro-apoptosis Better OS	The ability of PLAC2 to upregulate P53 and induce apoptosis in HCC cells leads to significantly higher OS rates in HCC patients with low levels of PLAC2.	[38]
lncRNA HCG18	↑GPX4	Anti-ferroptosis Poorer OS	Silencing HCG18 inhibits GPX4 by binding to miR-450b-5p, promotes GPX4-inhibited ferroptosis, and leads to a better prognosis in HCC patients	[39]
circRNA circ_0021093	↑MTA3	Anti-apoptosis Poorer OS	Downregulation of circ_0021093 markedly suppresses HCC cell proliferation through enhanced cell apoptosis, impaired migration, and invasion abilities, while overexpression of circ_0021093 predicts lower survival rates by targeting the miR-766-3p/MTA3 pathway in HCC.	[40]
circRNA circ-FOXP1	-	Anti-apoptosis Poorer OS	The circRNA circ-FOXP1 plays a cell-protective role by sequestering miR-875-3p and miR-421, thereby mitigating apoptosis. HCC patients with elevated circ-FOXP1 expression exhibited reduced OS rates.	[41]
circRNA circMDK	-	Anti-apoptosis Poorer OS	Knockdown of circRNA circMDK enhances the apoptosis ratio of HCC cells, suggesting its potential tumor suppressor role, while increased circMDK expression is associated with poorer 5-year survival probability in HCC patients.	[27]
m7G-tRNA	↑EGFR	Anti-apoptosis Poorer OS	METTL1/WDR4-mediated m7G tRNA modification in promoting translation of EGFR pathway genes to reduce apoptosis and trigger drug resistance in HCC cells	[43]
m1G37-tRNA	↑HIF-1	Anti-apoptosis Poorer OS	Knockdown of TRMT5 inactivated the HIF-1 signaling pathway by preventing HIF-1α stability through the enhancement of cellular oxygen content.	[44]

Abbreviations: ACSL4, acyl-CoA synthetase long-chain family member 4; DFS, disease-free survival; EGFR, epidermal growth factor receptor; EZH2, enhancer of zeste homolog 2; GPX4, glutathione peroxidase 4; HCC, hepatocellular carcinoma; HIF-1, hypoxia-inducible factor-1; MTA3, metastasis-associated protein 3; ncRNA, non-coding RNA; OS, overall survival; PFS, progression-free survival; RFS, relapse-free survival; TRMT5, tRNA methyltransferase 5; ↑, elevated expression level; ↓, decreased expression level.

## 3. Drug Resistance

### 3.1. Targeted Therapy and Drug Resistance

Targeted therapy is a widely recognized therapeutic approach that aims to selectively inhibit the activity of specific molecular pathways implicated in the proliferation and progress of tumors. Sorafenib and lenvatinib are two targeted agents that have demonstrated clinical efficacy in the first-line treatment of advanced HCC [62,63]. They belong to multi-kinase inhibitors that block multiple signaling pathways involved in tumor angiogenesis, proliferation, invasion, and metastasis [64,65]. Despite the advancements achieved in the area of multikinase inhibitors, which have indicated hope for the treatment of advanced HCC, a significant number of patients fail to receive therapeutic benefits from this approach. This is evidenced by the low response rates observed and the emergence of drug resistance. Currently, multiple mechanisms associated with resistance to targeted therapy have been elucidated. These mechanisms include epigenetics, transport processes, regulated cell death, and the tumor microenvironment. Most targeted therapies are designed to activate PCD pathways to eradicate tumor cells. However, in the case of cancer, the dysregulation of PCD signaling, specifically the impairment of anti-apoptotic mechanisms, results in unrestricted tumor growth and the emergence of resistance toward targeted therapeutic interventions [66]. The issue of drug resistance poses a significant obstacle in the treatment of HCC, thereby reducing the effectiveness of targeted therapies [67]. In accordance with previous investigations, ncRNAs have been recognized as pivotal modulators of PCD and have been linked to the development of resistance to targeted therapy in HCC (Table 2).

#### 3.1.1. Sorafenib Resistance (SR)

##### Apoptosis

The development of tumor cell apoptosis is a crucial mechanism underlying the therapeutic efficacy of sorafenib [101]. ncRNAs possess the ability to regulate pivotal signaling pathways implicated in apoptosis-related processes, thereby exerting an influence on the response to Sorafenib through these pathways. 

Protein kinase B (AKT), a serine–threonine kinase, is crucial in mediating diverse biological functions. In addition to its fundamental involvement in regular cellular physiology, numerous investigations have provided evidence for the activation of the AKT cascade in diverse forms of human cancer, frequently leading to heightened tumor aggressiveness and the development of resistance to therapeutic drugs. AKT inactivates pro-apoptotic proteins, such as BCL-2-antagonist of cell death and procaspase-9, to block apoptosis [102]. miR-92b has been identified as a regulator targeting the phosphatase and tensin homolog (PTEN) gene in HCC cells. This targeting event leads to the activation of the phosphatidylinositol 3 kinase (PI3K)/AKT/mammalian target of the rapamycin (mTOR) signaling pathway within these cells. Consequently, the activation of this pathway promotes cellular survival and proliferation in HCC cells. Following transfection with the miR-92b inhibitor, a notable attenuation in the proliferation of HepG2/SR cells was observed, accompanied by a significant elevation in apoptosis rates [68]. Similarly, the lncRNA SNHG1 activates the AKT pathway by regulating SLC3A2, and its inhibition enhances sorafenib-induced apoptosis. Another role of a lncRNA called NEAT1 is noted in the downregulation of miR-335 and its downstream effects on the c-Met–Akt pathway. The findings suggest that NEAT1 exerts a negative regulatory effect on miR-335, which subsequently leads to the suppression of the c-Met-Akt pathway. These results contribute to understanding the intricate molecular mechanisms involved in regulating cellular pathways and highlight the potential significance of NEAT1 in modulating miRNA-mediated signaling cascades [71]. The administration of sorafenib has been observed to elicit the translocation of miR-21 to the nucleus, thereby facilitating the upregulation of SNHG1 expression. At the same time, miR-21 can also reduce the expression of PTEN, resulting in the activation of the AKT pathway independent of SNHG1 [69]. 

In contrast, some ncRNAs can decrease SR by impeding AKT signaling. The suppression of RAF/ERK and PI3K/AKT signaling pathways is observed upon inhibition of the Kirsten rat sarcoma viral oncogene (KRAS). The KRAS gene has been identified as a direct target of miR-622. The combined inhibition of KRAS and sorafenib treatment has been shown to enhance the sensitivity of sorafenib-resistant cells to the effects of sorafenib [73]. lncRNA HEIH can act as a sponge for miR-98-5p, and its inhibition activates the PI3K/AKT pathway, enhancing SR in sorafenib-resistant HCC cells [74]. The continuous management of sorafenib has been observed to result in a reduction in the expression levels of circRNA ITCH in HCC cells. ITCH increases PTEN expression by sponging miR-20b-5p and then inactivates PI3K/Akt signals. In addition, the overexpression of ITCH was found to enhance the sensitivity to sorafenib, induce apoptosis, and reduce the migratory capacity of sorafenib-resistant HCC cells [75]. 

Furthermore, ncRNAs can modulate various targets, thereby influencing apoptosis and subsequently impacting SR. The overexpression of miR-486-3p induces apoptosis by targeting fibroblast growth factor receptor 4 (FGFR4) and epidermal growth factor receptor (EGFR). In an in vivo sorafenib-resistant model, lentivirus-mediated overexpression of miR-486-3p effectively overcame SR and remarkably suppressed tumor growth when combined with sorafenib treatment [76]. Similarly, the overexpression of miR-10b-3p has been observed to augment the induction of apoptosis via sorafenib in HCC cells. Conversely, the depletion of miR-10b-3p can partially eliminate this effect. Notably, miR-10b-3p exerts its influence by targeting cyclin E1, a downstream target [77]. In addition, lncRNA MALAT1 has been found to play a significant role in promoting the proliferation and migration of HCC cells while concurrently suppressing apoptosis. By acting as a molecular sponge for miR-140-5p, MALAT1 regulates the expression of Aurora-A, thereby promoting SR in HCC cells [72].

##### Autophagy

Autophagy represents a dualistic phenomenon in tumor drug resistance. The phenomenon under investigation contributes to the emergence of drug resistance in tumors, thereby shielding them from the effects of therapeutic drugs. It also exhibits the ability to eliminate drug-resistant tumor cells that possess non-functional apoptotic pathways. Furthermore, it may even facilitate the reversal of tumor drug resistance [103]. ncRNAs-regulated autophagy also plays an important role in SR.

lncRNA HANR has been identified as a significant factor in the development of SR in HepG2 and Huh7 cells. This resistance is believed to be facilitated by HANR’s promotion of autophagy. miR-29b can directly target autophagy-related gene 9A (ATG9A) and interact with HANR to counteract HANR-induced SR by suppressing autophagy in these cells [78]. In human HCC tissues, the expression of miR-25 is upregulated, and this upregulation is observed to be associated with the pathological grade of the tumor, clinical staging, and lymphatic metastasis. miR-25 enhances SR and autophagy in HCC cells by targeting FBXW7 protein, suggesting its potential as a novel therapeutic target for HCC treatment [79]. Sorafenib-resistant HCC cells demonstrate diminished susceptibility to sorafenib’s growth inhibitory and apoptotic effects, accompanied by heightened activation of AKT and its downstream effectors. The reduction of autophagy has been observed to decrease the susceptibility of resistant cells to sorafenib. The involvement of miR-21 in the development of acquired resistance to sorafenib is mediated by its inhibitory effect on autophagy via the PTEN/AKT pathway [70].

Interestingly, certain ncRNAs can enhance the sensitivity of HCC cells to sorafenib by inhibiting autophagy. For instance, the direct impact of miR-541 on ATG2A and Ras-related protein Rab-1B (RAB1B) was investigated to elucidate its role in inhibiting the malignant phenotype and autophagy of HCC cells. The potential of miR-541 expression as a predictive biomarker for the response to sorafenib treatment in HCC was noted exclusively. It indicates that higher levels of miR-541 expression are associated with a more favorable response to sorafenib therapy. Furthermore, the combination treatment of miR-541 and sorafenib exhibits superior inhibitory effects on HCC cell growth compared to treatment with sorafenib alone [80].

##### Ferroptosis

Ferroptosis has gained recognition as a widely observed and evolutionarily conserved mechanism of apoptosis. It involves the aberrant cellular metabolism of lipid oxidants, a process facilitated by iron ions or iron-containing enzymes. Sorafenib, a potent multikinase inhibitor, has been shown to exhibit tumor-inhibitory properties, at least partially through the induction of ferroptosis. Intriguingly, cancer cells that have acquired resistance to therapeutic interventions, particularly those in a mesenchymal state characterized by an increased likelihood of spreading to distant sites, demonstrate an augmented vulnerability to ferroptosis [104]. For example, metallothionein (MT)-1G knockdown has been shown to restore the anticancer activity of sorafenib by inducing iron death in HCC cells [105,106]. In addition, some ncRNAs may also influence SR by modulating ferroptosis. Furthermore, it has been observed that certain ncRNAs have the potential to affect cellular SR via their regulatory role in modulating the process of ferroptosis. A specific ferroptosis-associated lncRNA lncFAL, derived from the plexin B2 gene, can decrease the susceptibility to ferroptosis by directly interacting with a protein known as ferroptosis suppressor protein 1 (FSP1). lncFAL competitively inhibits the degradation of FSP1 through polyubiquitination mediated by Trim69. These findings shed light on the molecular mechanisms underlying the regulation of ferroptosis and provide insights into potential therapeutic targets for modulating this process. This action has been observed to attenuate the anti-cancer efficacy of ferroptosis inducers, including sorafenib [81]. In a similar vein, the phenomenon of ferroptosis has been observed to be commonly inhibited in HCC cells that have developed resistance to sorafenib treatment. Silence of the lncRNA HCG18 effectively promotes ferroptosis to overcome this resistance. The pro-ferroptotic impact of HCG18 silencing can be counteracted by overexpressing GPX4. Silencing HCG18 inhibits GPX4 by binding to miR-450b-5p, promotes GPX4-inhibited ferroptosis, and averts SR in HCC [39]. Moreover, decreasing ACSL4 significantly reduces sorafenib-induced lipid peroxidation and ferroptosis in HCC cells. Higher ACSL4 level indicates improved response to sorafenib treatment [82]. Interestingly, although high ACSL4 levels positively correlate with sorafenib sensitivity in vitro, reduced miR-211-5p expression leading to increased ACSL4 levels will promote tumor proliferation and invasion [107]. The specific mechanism and treatment selection remain to be further studied.

Currently, efforts have been undertaken to mitigate SR via the regulation of ncRNAs. The investigation conducted by Tang et al. demonstrated that an artificial lncRNA was synthesized and designated as AlncRNA. This approach effectively hinders the activities of various miRNAs, including miR-21, miR-153, and miR-216a, among others. Consequently, the downregulation of PTEN and the activation of AKT are impeded, leading to substantial inhibition of cell proliferation and the induction of apoptosis in sorafenib-resistant HCC. The potential of lncRNA to augment the effectiveness of sorafenib has been demonstrated in both in vitro and animal model studies [108]. Further research and clinical trials can be conducted to explore the full potential of AlncRNA-based interventions and their application in improving patient outcomes. Other information related to SR can be found in this review. Future investigations and clinical trials should be undertaken to fully elucidate the potential of AlncRNA-based interventions and their impact on enhancing patient outcomes. 

Additional details regarding SR can be accessed in this comprehensive review [101].

#### 3.1.2. Lenvatinib Resistance (LR)

Over the last ten years, sorafenib has been the only approved targeted systemic therapy for individuals afflicted with advanced unresectable HCC. However, the current landscape has been significantly altered by the introduction of lenvatinib, a multitargeted tyrosine kinase inhibitor [109,110]. As a novel first-line treatment option for HCC, lenvatinib has demonstrated non-inferior overall survival, improved progression-free survival, a higher response rate, and a better disease control rate compared to sorafenib in multiple clinical trials [111,112]. 

However, the current state of scientific literature on lenvatinib and its effects on LR is limited due to the relatively recent introduction of this medication. In a recent investigation by Yu et al. (2021), it was found that lenvatinib treatment induces upregulation of lncRNA MT1JP, which acts as a sponge for miR-24-3p, thereby releasing B-cell lymphoma-2 like 2 (BLC2L2), an anti-apoptotic protein, to inhibit lenvatinib-induced cell apoptosis and form a positive feedback loop, leading to LR [83]. In 2023, a study discovered that METTL1 and WDR4 can form a stable protein complex to catalyze tRNA m7G modification. METTL1-mediated m7G tRNA modification enhances the translation of EGFR, thereby promoting the proliferative ability of HCC cells and inhibiting apoptosis to maintain resistance to lenvatinib in HCC [43]. With the increasing prevalence of lenvatinib utilization, the challenge of treatment resistance assumes a progressively severe nature. There is considerable evidence that ncRNAs can regulate HCC drug efflux and metabolism, glucose metabolism, malignant phenotypes, and cell death pathways [113,114,115]. PCD, as one of the anti-cancer mechanisms of lenvatinib, plays a significant role in LR [116,117].

### 3.2. Immune Therapy Drug Resistance

The liver can initiate immune responses to safeguard against the incursion of undesirable pathogens and the formation of tumors. However, in the case of HCC, which is frequently linked to chronic inflammation, the evasion of the immune system becomes a prominent characteristic during the initiation and progression of HCC [118]. Recently, there has been a notable surge of interest in the complex interaction between immune cells and tumor cells. The immune system is significantly influenced by various molecular mechanisms that contribute to the biological characteristics of tumor cells during the development of HCC [119]. Immunotherapy, as a burgeoning therapeutic modality, has exhibited substantial promise in the management of HCC. Among the various therapeutic approaches, immune checkpoint inhibitors (ICIs) have emerged as a promising strategy. Specifically, drugs targeting programmed cell death protein 1 (PD-1) and programmed cell death 1 ligand 1 (PD-L1) have demonstrated clinical efficacy. ICIs can obstruct the interaction between checkpoint proteins and their corresponding ligands. This obstruction prevents the deactivation of T cells and reinstates their capacity to identify and combat tumors. Consequently, ICIs exhibit an anti-tumor effect [120]. In 2020, the American Society of Clinical Oncology (ASCO) guidelines suggested the combination of ICI atezolizumab with the anti-vascular endothelial growth factor (VEGF) drug bevacizumab as a first-line systemic treatment for advanced unresectable HCC patients [121]. However, a considerable number of patients subjected to these pharmacological interventions demonstrated limited clinical response and sometimes developed drug resistance, thereby resulting in the advancement of the disease and eventual mortality of the patient.

The resistance to ICIs can be broadly categorized into two types: primary drug resistance and acquired drug resistance. In contrast to the extensive research conducted on primary drug resistance, there is a lack of studies and published literature on the acquired drug resistance of ICIs. Primary resistance, in the context of ICIs, typically pertains to patients who exhibit no response to ICIs and instead experience rapid disease progression or eventual treatment failure [122].

Currently, a recognized mechanism associated with primary resistance in cancer is the tumor mutational burden, which correlates with the efficacy of anti-PD-1 therapy. A higher tumor mutational burden indicates a better response to therapy in different types of tumors [123]. In contrast, the perturbation of crucial signaling pathways within tumors can also result in primary resistance towardICIs. Interferon-gamma (IFN-γ), a crucial cytokine, plays a pivotal role in initiating and maintaining potent anti-tumor reactions. The compound augments the functionality of CD8+ cytotoxic T cells and demonstrates anti-proliferative and pro-apoptotic properties. Additionally, it induces the upregulation of major histocompatibility complex class I (MHC I) molecules in tumor cells. In the clinical setting, a robust IFN-γ signature has been recognized as a valuable prognostic marker and a reliable indicator of treatment response in patients with different cancer types undergoing anti-PD-1 therapy. Identification of intrinsic mutations within tumors that disrupt signaling in the IFN-γ pathway has been recognized as a pivotal factor in conferring resistance to ICIs [124].

The effectiveness of ICIs is intricately linked to the condition of the tumor immune microenvironment. The efficacy of anti-tumor immunotherapy is primarily contingent upon the condition and functionality of immune cells present within the tumor microenvironment [125] (Table 2). Tim-3, an immune checkpoint that negatively regulates T cell-dependent immune responses, has been extensively investigated in previous studies due to its role in the negative regulation of immune responses mediated by T cells [126]. A study noted that upregulation of lncRNA NEAT1 in peripheral blood monocytes of HCC patients can interfere with the expression of Tim-3 by binding to miR-155. Downregulation of NEAT1 inhibits CD8+ T cell apoptosis and enhances cytotoxic activity, thereby suppressing tumor growth [84]. Moreover, high expression of circSOD2 in HCC cellspromotes cancer cell migration and invasion while inhibiting apoptosis of cancer cells. This state of high expression also results in the apoptosis of CD8+ T cells, causing dysfunction of CD8+ T cells and promoting immune escape in HCC, thereby inhibiting the therapeutic efficacy of anti-PD-1 drugs. The axis of circSOD2/miR-497-5p/ Annexin A11 (ANXA11) is associated with this effect [85].

Tumor cells can exhibit an excessive expression of PD-L1, a protein that interferes with the signaling pathway of T-cell receptors. This interference leads to functional impairment and/or apoptosis of T cells expressing PD-1 receptors [127,128]. The intensity of PD-L1 expression on tumor cells is a frequently utilized biomarker in the context of ICIs resistance. This is not only because it ensures the presence of targetable PD-1 receptor-ligand interactions in the tumor microenvironment at a fundamental level, but also because PD-L1 expression is associated with immune activation, including CD8+ T-cell response and antigen presentation [129]. ncRNAs can regulate the expression of PD-L1, thus influencing the immunotherapy’s effect (Table 2). In HCC cells, downregulation of miR-675-5p can enhance the stability of PD-L1 mRNA, possibly leading to the accumulation of PD-L1 in HCC cells by affecting the 3’-untranslated region of PD-L1 [86]. miR-378a-3p levels also show a downward trend in HCC and are negatively correlated with PD-L1 levels [87]. Additionally, certain ncRNAs can regulate the expression of PD-L1 on HCC cells by modulating signal transducers and activators of transcription (STAT) family proteins. For instance, it has been discovered that the lncRNA MIR155HG functions as a sponge for miR-233, thereby upregulating PD-L1 expression through the miR-223/STAT1 axis [88]. Moreover, lncRNA MIAT has been found to negatively regulate miR-411-5p, leading to an upregulation of STAT3 and ultimately increasing PD-L1 expression at the transcriptional level [89].

Furthermore, ncRNAs can also regulate the expression of PD-L1 on macrophages. An intriguing phenomenon was observed by Liu et al. in HCC cells under endoplasmic reticulum stress: HCC cells can transfer miR-23a-3p to macrophages through extracellular vesicles, thereby influencing the expression of PD-L1 in macrophages. Specifically, extracellular vesicle-derived miR-23a-3p inhibits the expression of PTEN within macrophages, activating the PI3K/AKT pathway and upregulating PD-L1 expression in macrophages. This further leads to increased apoptosis of T cells and a decrease in the proportion of CD8+ T cells, inducing an immunosuppressive effect [90]. Some other ncRNAs that have regulatory effects on PD-L1 can be seen in Table 2 [91,92,93,94,95].

Despite the emerging potential of immunotherapy as a therapeutic modality in the realm of HCC, it remains encumbered by inherent limitations. In light of the growing recognition of the pivotal involvement of ncRNAs in the immune evasion mechanisms employed by liver cancer cells, investigators have the opportunity to investigate potential interventions aimed at modulating these ncRNAs. This can be achieved through the utilization of various approaches, including the utilization of miRNA mimics, small molecules, or gene editing techniques, to effectively regulate the expression levels of these ncRNAs. The ultimate goal of such interventions would be to augment the effectiveness of immunotherapy strategies in the context of HCC. Moreover, considering the heterogeneity of HCC patients and the intricate nature of the immune system, future investigations must prioritize the development of individualized immunotherapeutic approaches. This requires exploring the potential application of ncRNAs in the regulation of the PD-1/PD-L1 signaling pathway and other immune checkpoints. Additionally, the synergistic effects of combining ncRNA mimics or inhibitors with anti-PD-1/PD-L1 drugs or other immunotherapeutic agents should be investigated to enhance the efficacy and safety of immunotherapy specifically for liver cancer.

### 3.3. Chemotherapy Resistance

Chemotherapy continues to play a significant role in the therapeutic management of HCC [130]. Transcatheter arterial chemoembolization TACE has emerged as the preferred therapeutic approach for managing HCC at the intermediate stage. Meanwhile, extensively utilized in Asia, hepatic artery infusion chemotherapy has been incorporated into treatment guidelines in Japan and South Korea [131,132]. Chemotherapy resistance frequently results in treatment ineffectiveness in HCC, wherein PCD regulated by ncRNAs assumes a significant role (Table 2). For instance, it has been demonstrated that miR-223 exerts inhibitory effects on doxorubicin (DOX)-induced autophagy in HCC cells by targeting forkhead box protein 3a (FOXO3a). This regulatory mechanism of miR-223 is implicated in the development of DOX resistance in HCC cells [96]. Another miRNA, microRNA-200a-3p enhances 5-fluorouracil (5-FU) resistance by regulating dual specificity phosphatase 6 (DUSP6) expression [97]. In addition, it has been observed that the long intergenic non-protein coding RNA 01134 (LINC01134) plays a role in activating the anti-oxidative pathway by facilitating the recruitment of the transcription factor SP1 to the promoter region of p62. This activation of the SP1/P62 pathway by LINC01134 reduces cell apoptosis and consequently enhances the resistance of HCC cells to oxaliplatin, a chemotherapeutic agent [98]. The findings from the study conducted by Li et al. revealed that circARNT2 acts as a competitive molecule against miR-155-5p, leading to the upregulation of PDK1-induced autophagy. This mechanism ultimately results in an augmented sensitivity of HCC cells to cisplatin, enhancing its therapeutic effectiveness [99]. In contrast, HCC cells that exhibit overexpression of circular RNA circMRPS35 demonstrate a reduced rate of apoptosis. The role of MRPS35 as a ceRNA for miR-148a-3p leads to the modulation of syntaxin 3 (STX3) expression, thereby causing its upregulation. The STX3 protein is responsible for initiating the process of ubiquitination and subsequent degradation of the PTEN protein. This mechanism ultimately leads to a decrease in apoptosis and an increase in resistance to cisplatin [100].

## 4. Drug Side Effects

**Figure 3:**
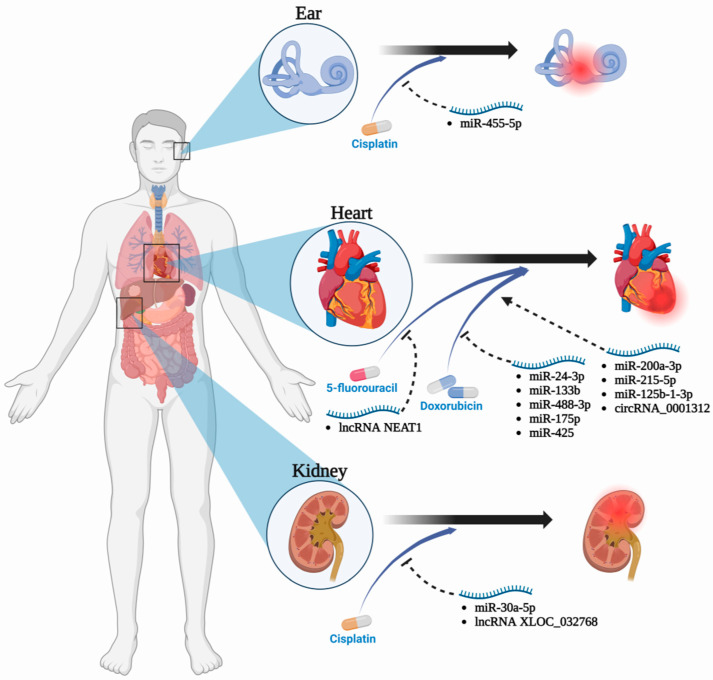
The regulation of chemotherapy drug side effects by ncRNAs-regulated programmed cell death.

Drug-induced side effects are frequently observed in systemic treatment regimens. Presently, there is a growing body of research investigating the influence of ncRNAs on the occurrence and severity of chemotherapy-related side effects [133]. Whether systemic chemotherapy or interventional administration is employed, chemotherapeutic drugs can potentially induce toxic side effects, causing patients to experience treatment intolerance. In response to these challenges, researchers have endeavored to discover treatments that are both more efficacious and better tolerated. PCD plays a significant role as one of the key mechanisms underlying the side effects of chemotherapy drugs. Understanding the regulation of PCD by ncRNAs is crucial in the search for more effective and better-tolerated treatments (Figure 3, Table S1).

### 4.1. Anthracycline

Anthracyclines, such as DOX, have been widely recognized for their exceptional therapeutic effectiveness in the management of various types of cancers, including HCC. However, the clinical utility of these compounds is constrained by their propensity to elicit life-threatening cardiotoxic effects, including the development of irreversible degenerative dilated cardiomyopathy and congestive heart failure [134]. DOX-induced cardiac toxicity has been extensively studied, with apoptosis identified as the primary cellular process involved in cardiac cells [135,136]. Multiple ncRNAs have been identified as regulators of signaling pathways implicated in cellular apoptosis. These ncRNAs play a crucial role in modulating the response of cardiac cells to DOX and regulating the magnitude of cardiac injury.

In the context of DOX, it has been observed that some ncRNAs exhibit an increased expression level. These ncRNAs are crucial elements that facilitate apoptosis in cardiac cells. The expression of miR-200a-3p is increased in the myocardial injury model induced by DOX. The administration of an miR-200a-3p inhibitor demonstrates partial mitigation of DOX-induced cardiac toxicity in a rat model. miR-200a-3p has been identified as a regulator of paternally expressed gene 3 (PEG3) and is involved in the modulation of myocardial cell proliferation and apoptosis. This regulatory mechanism operates through the Sirtuin 1 (SIRT1)/nuclear factor kappa B (NF-κB) signaling pathway [137]. In addition to miR-200a-3p, miR-215-5p also plays an important role in the process of DOX treatment. miR-215-5p can downregulate the expression of zinc finger E-box-binding homeobox 2 (ZEB2), thereby inducing apoptosis in myocardial cells [138]. In recent investigations, it has been discovered that circRNA_0001312 exhibits the ability to sequester miR-409-3p via the ceRNA mechanism. miR-409-3p can target high-mobility group box 1 (HMGB1), thereby reducing DOX-induced myocardial cell apoptosis. This further confirms the regulatory role of circRNA_0001312 in the process of DOX-induced myocardial cell injury [139].

In contrast, some ncRNAs exhibit downregulation upon treatment with DOX, thereby exerting a protective influence on cardiac cells, mitigating potential damage. Exposure of cardiomyocytes to DOX induces oxidative stress, thereby causing a subsequent elevation in intracellular levels of reactive oxygen species (ROS), which catalyzes the initiation of cellular apoptosis. miR-24-3p can inhibit this process by activating the nuclear factor erythroid 2-related factor 2 (Nrf2) pathway [140,141]. Furthermore, the levels of miR-133b are decreased in HL-1 cardiomyocytes and mouse hearts after DOX treatment. miR-133b can alleviate DOX-induced myocardial cell apoptosis and cardiac fibrosis by targeting polypyrimidine tract binding protein 1 (PTBP1) and transgelin 2 (TAGLN2) [142]. Simultaneously, miR-488-3p is also a crucial protective ncRNA in DOX-induced cardiotoxicity. It promotes the viability of cardiac cells and attenuates cardiomyocyte autophagic flux blockage and apoptosis by inhibiting cyclin G1 [143].

As the only Food and Drug Administration(FDA)-approved cardioprotective drug for preventing DOX toxicity, dexrazoxane can increase the levels of miR-175p in cardiac myocytes, which can target and reduce the expression of PTEN, thereby attenuating DOX-induced cardiomyocytes apoptosis [144]. However, dexrazoxane may reduce the anti-cancer effects of anthracycline drugs and cause severe adverse reactions [145]. Some researchers have been exploring new drugs that intervene with ncRNAs to improve the cardiac toxicity associated with DOX therapy. For instance, Li et al. explored that rutin has the ability to activate the JunD signaling pathway, reduce the levels of miR-125b-1-3p, and decrease the expression of certain apoptotic proteins, such as p21, cleaved caspase-3, and cleaved caspase-9. It can significantly enhance the survival rate of cardiomyocytes under the influence of pirarubicin [146]. Guo et al. identified Irisenin, an isoflavonoid isolated from the rhizome of Belamcanda chinensis, can activate miR-425, thereby reducing the expression of receptor-interacting protein kinase 1 (RIPK1) and alleviating apoptosis in HL-1 cells induced by DOX [147]. With an increasing number of drugs being discovered to alleviate DOX-induced cardiotoxicity, researchers can compare different ncRNA intervention strategies in terms of inhibiting cardiac toxicity and maintaining anti-tumor effects in order to identify the optimal combination of approaches and dosages. This exploration will contribute to the development of more effective treatment methods to mitigate the adverse impact of DOX-induced cardiac toxicity on patients.

### 4.2. Platinum Drugs

Cisplatin, a widely employed platinum-based antineoplastic agent, has therapeutic effects by impeding DNA synthesis through direct covalent interaction with DNA molecules [148]. In managing advanced HCC, the utilization of cisplatin in conjunction with other pharmacological agents has yielded a notable objective response rate of 36% [149]. However, the side effects of cisplatin on normal tissues and organs, particularly kidney tissue damage characterized by tubular cell necrosis and apoptosis, limit its clinical use [150]. To address this matter, recent investigations have directed their attention toward exploring the potential of ncRNAs in modulating the adverse effects associated with cisplatin therapy. Among them, a lncRNA called XLOC_032768 reduces the expression of tumor necrosis factor-alpha (TNF-α) induced by cisplatin in renal tubular epithelial cells through a transport mechanism. Research indicates that inhibiting the expression of TNF-α can improve cisplatin-induced apoptosis of renal tubular epithelial cells and kidney structural damage [151]. Additionally, in a study investigating the protective effects of extracellular vesicles in premature infant urine against cisplatin-induced acute kidney injury, researchers found that miR-30a-5p, the most abundant miRNA in premature infant urine-derived extracellular vesicles, can target and downregulate mitogen-activated protein kinase 8 (MAPK8) to protect HK-2 cells from cisplatin-induced apoptosis [152]. Besides nephrotoxicity, the regulation of ncRNAs can also alleviate cisplatin-induced ototoxicity. Studies have shown that resveratrol can upregulate the expression of miR-455-5p, thereby reducing PTEN levels and activating the PI3K/AKT pathway. This increases the survival rate of cisplatin-induced HEI-OC1 cells and reduces apoptosis and oxidative stress [153].

Despite the current limited research, ncRNAs have shown potential importance in regulating the side effects of cisplatin treatment. Further investigations can delve into the interactions between ncRNAs and cisplatin and explore how these ncRNAs can be utilized to alleviate the side effects associated with cisplatin therapy. This will provide novel therapeutic strategies for improving the efficacy of cisplatin and reducing its adverse effects.

### 4.3. Fluorouracil

Additionally, 5-FU and other fluoropyrimidine drugs have emerged as fundamental components of diverse chemotherapy regimens, and 5-FU is widely recognized as one of the most frequently employed chemotherapeutic agents globally, specifically targeting the management of solid malignancies. However, 5-FU is also recognized as the second most prevalent chemotherapeutic agent linked to cardiotoxicity, following anthracyclines. Currently, our understanding of the mechanisms underlying 5-FU-induced cardiotoxicity is still limited [154]. Several studies have suggested that the lncRNA NEAT1 plays a crucial role in 5-FU treatment. NEAT1 acts as an endogenous competitor that can bind to miR-150-5p, and 5-FU can reduce the expression of NEAT1 while increasing the levels of miR-150-5p. In light of subsequent investigations, it has been discovered that the upregulation of miR-150-5p leads to the suppression of cleavage and polyadenylation-specific factor 4 (CPSF4) expression, thereby inducing apoptosis in tumor cells and inhibiting their proliferation [155,156]. However, the downregulation of NEAT1 expression results in an upregulation of miR-142-3p levels, consequently causing a decrease in the expression of FOXO1. This alteration can potentially contribute to the progression of cardiotoxicity and the induction of cardiomyocyte apoptosis [157]. In the future, the development of regulatory strategies targeting NEAT1, miR-150-5p, or FOXO1, such as the use of NEAT1 or FOXO1 mimics or antagonists, may help alleviate the cardiotoxicity associated with 5-FU treatment, thereby improving treatment efficacy and quality of life for patients.

## 5. Challenges in RNA Therapeutic Delivery

The effective transportation of RNA therapeutics to specific target cells and their subsequent internalization within the cells present notable obstacles in the scientific domain. To attain efficacious RNA-based therapeutic outcomes, it is imperative to surmount the obstacles linked to cellular and intracellular delivery [158].

At the subcellular level, the successful administration of RNA-based therapeutics encounters barriers associated with the inherent instability of RNA molecules and the ability to traverse the cell membrane effectively. The hindered diffusion of RNA molecules across cell membranes can be attributed to their hydrophilic nature and negative charge [159]. To address these challenges, researchers have explored a variety of strategies. By using cell-penetrating peptides (CPPs), foreign substances, such as chemicals, nucleic acids, and macromolecules, can pass through cell membranes and enter cells more effectively. CPPs have various sequence structures and basic mechanisms of action that have been elucidated. Among them, peptides rich in arginine have a distinct ability to cross the cellular barrier. These peptides possess essential amino acids with positive charges, enabling the formation of non-covalent interactions with drug molecules and cell membranes characterized by a negative charge [160]. In addition, antisense oligonucleotides formed by third-generation antisense nucleic acid technology are highly stable and, although difficult to take up by cells, can be absorbed through different endocytosis mechanisms using delivery systems, such as lipid- and polymer-based vectors and ligand oligonucleotide conjugates [161].

Upon entry into the specific cellular entity, RNA therapeutics encounter various intracellular challenges, specifically those associated with endosomal entrapment and intracellular transportation. Following cellular internalization, RNA molecules have the potential to undergo entrapment within the biological system, leading to their degradation or impaired release into the cytoplasm. To enhance intracellular escape, the prevailing strategy involves the utilization of endosomolytic agents. The translocation domain of diphtheria toxin, produced by *Corynebacterium diphtheriae*, is an extensively investigated fusogenic peptide. The conformational changes of diphtheria toxin are influenced by pH and facilitated by its translocation domain, enabling the toxin to cross the endosomal membrane and facilitate translocation into the cytoplasm [162]. Furthermore, incorporating fusogenic lipids into lipid-based nanocarriers can also improve the endosomal release of encapsulated nucleic acids [163].

In the intricate realm of RNA therapy challenges, if a domain is comparatively amenable for investigation, it would pertain to ailments linked with the hepatic system. The liver serves as a vital clearance organ, perpetually engaged in the filtration of various substances from the bloodstream. In addition, hepatocytes possess low-density lipoprotein receptors, which can be effectively used to transport a substantial quantity of RNA molecules into the cells. This approach has been applied in both lipid nanoparticles and naked RNA formulations [164]. Hence, within the domain of liver disorders, a multitude of prospects arise in the realm of RNA-based therapeutic interventions. With persistent research endeavors and pioneering advancements, there exists a promising prospect of enhancing the efficiency of RNA therapy delivery systems, unearthing novel therapeutic targets, and furnishing liver disease patients with enhanced treatment strategies that are both efficacious and safe.

## 6. Conclusions

ncRNAs are known to influence various cellular mechanisms, encompassing the intricate control of PCD. Their involvement in HCC holds substantial implications for identifying and predicting disease outcomes, drug resistance patterns, and the emergence of associated adverse effects. The process of PCD is a highly controlled mechanism that plays a crucial role in maintaining tissue homeostasis and removing unnecessary or impaired cells. The aberrant regulation of ncRNAs in HCC implies their potential involvement in the underlying disease mechanisms. Changes in the expression patterns of these ncRNAs can serve as diagnostic indicators in HCC, facilitating early identification and enhancing patient prognosis. In addition to their diagnostic use, ncRNAs have demonstrated their prognostic significance in HCC. Specific aberrant ncRNA expression correlates with HCC progression and patient survival, reflecting the invasiveness of the disease and influencing patient prognosis. These ncRNAs provide important insights into the molecular mechanisms underlying the development of HCC and can be used to develop personalized treatment strategies.

Furthermore, ncRNAs are implicated in HCC drug resistance and adverse effects, which continue to be significant obstacles to cancer treatment. They can modulate the signaling pathways of PCD, thereby increasing tumor sensitivity to chemotherapy, targeted therapy, or immunotherapy and reducing post-treatment adverse effects. Targeting ncRNA-regulated PCD to modulate drug resistance and associated side effects in HCC patients has been suggested by researches on drugs or drug carriers related to ncRNA.

In future research, further exploration of ncRNA functions and mechanisms, along with validation of their potential as targets in diagnosis, prognosis, and adjuvant therapy, is crucial. Simultaneously, addressing the challenges of targeted ncRNA therapy is essential. Targeting ncRNAs as therapeutic strategies holds tremendous potential in HCC patients, but effective delivery of therapeutic molecules to tumor cells remains a challenge. Therefore, efforts should be focused on developing novel delivery systems and technologies to achieve precise and efficient ncRNA delivery, ensuring specific accumulation in diseased tissues. Additionally, integrating ncRNA with other omics data and clinical information can help determine the optimal ncRNA application strategies in the personalized treatment of hepatocellular carcinoma. Such research can further improve patient prognosis and treatment outcomes, providing them with better quality of life and survival opportunities.

## Figures and Tables

**Table 2:** ncRNAs and drug resistance.

Therapy	ncRNAs	Targets	Effect	Mechanism	Refs.
Sorafenib	miR-92b	↓PTEN	Anti-apoptosis Promote SR	miR-92b reduces sorafenib-induced apoptosis and promotes SR by targeting PTEN and activating the PI3K/AKT/mTOR pathway.	[68]
	miR-21	↓PTEN	Anti-apoptosis Anti-autophagy Promote SR	miR-21 promotes the expression of SNHG1 while simultaneously downregulating the expression of PTEN, which leads to the activation of the AKT pathway, independent of SNHG1.	[69,70]
	lncRNA SNHG1	↑SLC3A2	Anti-apoptosis Promote SR	SNHG1 activates the AKT pathway by regulating SLC3A2, and its depletion enhances sorafenib-induced apoptosis.	[69]
	lncRNA NEAT1	↑c-Met-AKT	Anti-apoptotic Promote SR	NEAT1 can negatively regulate miR-335, which further suppresses the c-Met-AKT pathway.	[71]
	lncRNA MALAT1	↑Aurora-A	Anti-apoptosis Promote SR	MALAT1 regulated Aurora-A expression by sponging miR-140-5p, thus promoting SR in HCC cells.	[72]
	miR-622	↓KRAS	Pro-apoptosis Reduce SR	KRAS, which leads to the suppression of RAF/ERK and PI3K/AKT signaling pathways, is directly targeted by miR-622.	[73]
	lncRNA HEIH	↓PI3K/AKT	Pro-apoptotic Reduce SR	HEIH can act as a sponge for miR-98-5p, and its inhibition activates the PI3K/AKT pathway, promoting SR.	[74]
	circRNA ITCH	↑PTEN	Pro-apoptotic Reduce SR	ITCH increases PTEN expression by sponging miR-20b-5p and then inactivates PI3K/Akt signals.	[75]
	miR-486-3p	↓FGFR4 ↓EGFR	Pro-apoptotic Reduce SR	miR-486-3p induces apoptosis by targeting FGFR4 and EGFR, effectively overcoming SR and inhibiting tumor growth when combined with sorafenib treatment.	[76]
	miR-10b-3p	↓CyclinE1	Pro-apoptotic Reduce SR	Overexpressing miR-10b-3p enhances sorafenib-induced apoptosis in HCC cells, while depletion of miR-10b-3p partially abrogates this effect.	[77]
	lncRNA HANR	↑ATG9A	Pro-autophagy Promote SR	HANR promotes autophagy and contributes to SR. miR-29b targets ATG9A and counteracts HANR-induced SR by suppressing autophagy.	[78]
	miR-25	↓FBXW7	Pro-autophagy Promote SR	By inducing autophagy, miR-25 enhances SR in HCC and concurrently downregulates the expression of FBXW7 protein to modulate autophagy.	[79]
	miR-541	↓ATG2A ↓RAB1B	Anti-autophagy Reduce SR	miR-541 directly acts on ATG2A and RAB1B, thereby inhibiting the malignant phenotype and autophagy of HCC cells. Higher miR-541 expression predicts a better response to sorafenib.	[80]
	lncRNA lncFAL	↑FSP1	Anti-ferroptosis Promote SR	lncFAL reduces ferroptosis vulnerability by directly binding to FSP1 and competitively inhibiting Trim69-mediated FSP1 polyubiquitination degradation, thereby diminishing the anti-cancer effect of ferroptosis inducers like sorafenib.	[81]
	lncRNA HCG18	↑GPX4	Anti-ferroptosis Promote SR	Silencing HCG18 inhibits GPX4 by binding to miR-450b-5p, promotes GPX4-inhibited ferroptosis, and averts SR in HCC.	[39]
	miR-211-5p	↓ACSL4	Anti-ferroptosis Promote SR	Decreasing ACSL4 significantly reduces sorafenib-induced lipid peroxidation and ferroptosis in HCC cells. Higher ACSL4 level indicates improved response to sorafenib treatment	[82]
Lenvatinib	lncRNA MT1JP	↑BCL2L2	Anti-apoptosis Promote LR	The inhibition of apoptosis by the MT1JP-mediated miR-24-3p/BCL2L2 axis promotes resistance to lenvatinib in HCC cells.	[83]
	m7G-tRNA	↑EGFR	Anti-apoptosis Promote LR	METTL1/WDR4-mediated m7G tRNA modification in promoting translation of EGFR pathway genes to reduce apoptosis and trigger drug resistance in HCC cells.	[43]
Immune therapy	LncRNA NEAT1	↓Tim3	Promote CD8+ T cell apoptosis	The increased expression of lncRNA NEAT1 in peripheral blood monocytes of HCC patients can disrupt the expression of Tim-3 by interacting with miR-155. The reduction of NEAT1 suppresses apoptosis in CD8+ T cells.	[84]
	circRNA circSOD2	↑ANXA11	Promote CD8+ T cell apoptosis	High circSOD2 expression induces CD8+ T cell death, leading to CD8+ T cell dysfunction and immune escape in HCC, thereby hindering the effectiveness of anti-PD-1 drugs.	[85]
	miR-675-5p	-	↓PD-L1	Downregulating miR-675-5p stabilizes PD-L1 mRNA, leading to the accumulation of PD-L1 in HCC cells.	[86]
	miR-378a-3p	-	↓PD-L1	PD-L1 3′-UTR is a target of miR-378a-3p. miR-378a-3p suppresses PD-L1 expression in HCC cells.	[87]
	lncRNA MIR155HG	↑STAT1	↑PD-L1	MIR155HG functions as a sponge for miR-233, thereby upregulating PD-L1 expression through the miR-223/STAT1 axis.	[88]
	lncRNA MIAT	↑STAT3	↑PD-L1	MIAT negatively regulates miR-411-5p, leading to an upregulation of STAT3 and ultimately increasing PD-L1 expression at the transcriptional level	[89]
	mir-23a-3p	↓PTEN ↑PI3K/AKT	↑PD-L1 (Macrophage)	Extracellular vesicle-mediated transfer of miR-23a-3p from HCC cells to macrophages upregulates PD-L1 expression by inhibiting PTEN and activating the PI3K-AKT pathway. This results in increased T cell apoptosis and decreased CD8+ T cell proportion.	[90]
	miR-513a-5p	-	↓PD-L1	GUSB downregulates PD-L1 expression by promoting miR-513a-5p. GUSB inhibitor can improve the sensitivity of anti-PD1 therapy.	[91]
	lncRNA LINC00657	↓miR-424	↑PD-L1	LINC00657 exerts regulatory control over PD-L1 expression by acting as a miR-424 sponge, consequently influencing the progression of HCC.	[92]
	lncRNA LINC00244	-	↓PD-L1	LINC00244 downregulates PD-L1 and suppresses cell growth and metastasis in HCC.	[93]
	lncRNA HOXA-AS3	↓miR-455-5p	↑PD-L1	HOXA-AS3 increased the expression of PD-L1 by sponging miR-455-5p.	[94]
	circRNA circWDR25	↑ALOX15	↑PD-L1	circWDR25 regulates the expression of ALOX15 by sponging miR-4474-3p, ultimately inducing an epithelial-to-mesenchymal transition, and then promoting the expression of PD-L1 in HCC cells.	[95]
Doxorubicin	miR-223	↓FOXO3a	Anti-autophagy Promote resistance	miR-223 can inhibit DOX-induced autophagy by targeting FOXO3a and contributes to DOX-resistance in HCC cells.	[96]
5-fluorouracil	miR-200a-3p	↓DUSP6	Anti-apoptosis Promote resistance	microRNA-200a-3p increases 5-fluorouracil resistance by regulating DUSP6 expression.	[97]
Oxaliplatin	lncRNA LINC01134	↑P62 ↑LSD1	Anti-apoptosis Promote resistance	LINC01134 reduces cell apoptosis through the SP1/P62 pathway, thereby enhancing oxaliplatin resistance in HCC.	[98]
Cisplatin	circRNA circARNT2	↑PDK1	Pro-autophagy Reduce resistance	circARNT2 functions as a competing molecule against miR-155-5p, resulting in the upregulation of PDK1-induced autophagy and increasing sensitivity of HCC cells to cisplatin.	[99]
	circRNA circMRPS35	↑STX3	Anti-apoptosis Promote resistance	circMRPS35 acts as a sponge for miR-148a-3p, resulting in the upregulation of STX3 expression. STX3, in turn, induces the ubiquitination and degradation of PTEN to reduce apoptosis and promote cisplatin resistance.	[100]

Abbreviations: 3′UTR, 3′-untranslated region; ACSL4, acyl-CoA synthetase long-chain family member 4; AKT, protein kinase B; ALOX15, Arachidonate-15-Lipoxygenase; ANXA11, Annexin A11; ATG, autophagy-related gene; BLC2L2, B-cell lymphoma-2 like 2; circRNA, circular RNA; DOX, doxorubicin; DUSP6, dual specificity phosphatase 6; EGFR, epidermal growth factor receptor; FGFR4, fibroblast growth factor receptor 4; FOXO3a, forkhead box protein 3a; FSP1, ferroptosis suppressor protein 1; GPX4, glutathione peroxidase 4; GUSB, β-Glucuronidase; HCC, hepatocellular carcinoma; KRAS, Kirsten rat sarcoma viral oncogene; lncRNA, long non-coding RNA; LR, lenvatinib resistance; mTOR, mammalian target of rapamycin; ncRNA, non-coding RNA; PD-L1, programmed cell death 1 ligand 1; PI3K, phosphatidylinositol 3 kinase; RAB1B, Ras-related protein Rab-1B; SR, sorafenib resistance; STAT, signal transducers and activators of transcription. STX 3, syntaxin 3; ↑, elevated expression level; ↓, decreased expression level.

## Supplementary Materials

The following supporting information can be downloaded at: https://www.mdpi.com/article/10.3390/cancers15164187/s1, Table S1: ncRNAs and drug side effects.
