# Supplementary material for: Clinical Significance of Non-Coding RNA Regulation of Programmed Cell Death in Hepatocellular Carcinoma
Source: Cancers (Basel). 2023 Aug 21;15(16):4187. doi: 10.3390/cancers15164187 (PMC10452865; doi:10.3390/cancers15164187)
Supplement: Supplementary file 1 [file cancers-15-04187-s001.zip › cancers-2487692-supplementary.pdf]

Table S1. ncRNAs and drug side effects.

Drug	ncRNAs	Targets	Effect	Mechanism	Refs.
Anthracycline	miR-200a-3p	↓ PEG3	Pro-apoptosis Promote cardiotoxicity	MiR-200a-3p targets PEG3. Inhibition on miR-200a-3p leads to increased SIRT1 expression level while suppresses the NF-κB activity and reduces cell apoptosis.	[132]
	miR-215-5p	↓ ZEB2	Pro-apoptosis Promote cardiotoxicity	MiR-215-5p can downregulate the expression of ZEB2, thereby inducing apoptosis in myocardial cells.	[133]
	circRNA_0001312	↑ HMGB1	Pro-apoptosis Promote cardiotoxicity	CircRNA_0001312 can sponge miR-409-3p， which targets HMGB1, thereby reducing DOX-induced myocardial cell apoptosis.	[134]
	miR-24-3p	↑ Nrf2	Anti-apoptosis Reduce cardiotoxicity	MiR-24-3p can inhibit ROS induced cell apoptosis by activating Nrf2 pathway.	[135,136]
	miR-133b	↓ PTBP1<br>↓ TAGLN2	Anti-apoptosis Reduce cardiotoxicity	MiR-133b can alleviate DOX-induced myocardial cell apoptosis and cardiac fibrosis by targeting PTBP1 and TAGLN2.	[137]
	miR-488-3p	↓ CyclinG1	Anti-apoptosis Reduce cardiotoxicity	MiR-488-3p enhances the viability of cardiac cells and attenuates cardiomyocyte autophagic flux blockage and apoptosis by inhibiting Cyclin G1.	[138]
Cisplatin	lncRNA XLOC_032768	↓ TNF-α	Anti-apoptosis Reduce nephrotoxicity	XLOC_032768 reduces the expression of TNF-α induced by cisplatin and then improves cisplatin-induced apoptosis of renal tubular epithelial cells and kidney structural damage.	[146]
	miR-30a-5p	↓ MAPK8	Anti-apoptosis Reduce nephrotoxicity	MiR-30a-5p can target and downregulate MAPK8 to protect HK-2 cells from	[147]

				cisplatin-induced apoptosis.	
	miR-455-5p	↓ PTEN	Anti-apoptosis Reduce ototoxicity	MiR-455-5p suppresses PTEN levels and activates PI3K/AKT pathway. This reduces cisplatin-induced apoptosis and oxidative stress.	[148]
Fluorouracil	lncRNA NEAT1	↑ FOXO1	Anti-apoptosis Reduce cardiotoxicity	NEAT1 acts as an anti-apoptotic molecule by competitively binding with miR-142-3p as a ceRNA. The NEAT1/miR-142-3p/FOXO1 axis is involved in the cardioprotective mechanism, offering protection against cardiomyocyte apoptosis.	[152]

Abbreviations: AKT, protein kinase B; ceRNA, competing endogenous RNAs; circRNA, circular RNA; DOX, doxorubicin; FOXO1, forkhead box protein O1; HCC, hepatocellular carcinoma; HMGB1, high-mobility group box 1; lncRNA, long non-coding RNA; MAPK8, mitogen-activated protein kinase 8; ncRNA, non-coding RNA; NF-κB, Nuclear Factor Kappa B; Nrf2, nuclear factor erythroid 2-related factor 2; PEG3, paternally expressed gene 3; PI3K, phosphatidylinositol 3 kinase; PTBP1, polypyrimidine tract binding protein 1; ROS, reactive oxygen species; SIRT1, sirtuin 1; TAGLN2, transgelin 2; TNF-α, tumor necrosis factor-alpha; ZEB2, Zinc finger E-box-binding homeobox 2; ↑, elevated expression level; ↓, decreased expression level.
